# How *Drosophila melanogaster* Forms its Mechanoreceptors

**DOI:** 10.2174/138920208785133271

**Published:** 2008-08

**Authors:** D.P Furman, T.A Bukharina

**Affiliations:** 1 Institute of Cytology and Genetics, Siberian Branch, Russian Academy of Sciences, pr. Lavrentieva 10, Novosibirsk, 630090 Russia; 2 Novosibirsk State University, ul. Pirogova 2, Novosibirsk, 630090, Russia

**Keywords:** *Achaete–scute* complex, signaling pathways, macrochaetes, bristle pattern, drosophila.

## Abstract

A strictly determined number of external sensory organs, macrochaetes, acting as mechanoreceptors, are orderly located on drosophila head and body. Totally, they form the bristle pattern, which is a species-specific characteristic of drosophila.

Each mechanoreceptor comprises four specialized cells derived from the single sensory organ precursor (SOP) cell. The conserved bristle pattern combined with a comparatively simple structure of each mechanosensory organ makes macrochaetes a convenient model for studying the formation spatial structures with a fixed number of elements at certain positions and the mechanism underlying cell differentiation.

The macrochaete morphogenesis consists of three stages. At the first stage, the proneural clusters segregate from the massive of ectodermal cells of the wing imaginal disc. At the second stage, the SOP cell is determined and its position in the cluster is specified. At the third stage, the SOP cell undergoes two asymmetric divisions, and the daughter cells differentiate into the components of mechanoreceptor: shaft, socket, bipolar neuron, and sheath.

The critical factor determining the neural pathway of cell development is the content of proneural proteins, products of the *achaete-scute *(*AS-C*) gene complex, reaching its maximum in the SOP cell.

The experimental data on the main genes and their products involved in the control of bristle pattern formation are systematized. The roles of *achaete-scute* complex, EGFR and Notch signaling pathways, and selector genes in these processes are considered. An integral scheme describing the functioning of the system controlling macrochaete development in *D. melanogaster* is proposed based on analysis of literature data.

## INTRODUCTION

Bristles (mechanoreceptors), whose total number reaches 6000, are a component of the drosophila peripheral nervous system.

The bristles are divided into macro- and microchaetes according to their size and location on the fly head and body. These two types of mechanoreceptors display certain morphological and functional differences but the same developmental patterns and genetic control [1, 2].

Microchaetes (small bristles) are numerous and have no strictly determined localization, being organized in more or less regular rows. Unlike microchaetes, the number and location of macrochaetes (large bristles) are stringently determined, representing a species-specific drosophila characteristic, which makes them a classification criterion [2]. The bristle pattern characteristic of each species had been evolutionarily established by losing part of macrochaetes from the common ancestral set [1-3]. In particular, the bristle pattern of *D. melanogaster* is formed by 11 pairs of macrochaetes. Their positions are so constant that each bristle got its individual name depending on the position.

The bristle organ comprises the shaft, socket around its base, bipolar neuron, and neuron sheath. The shaft and socket are well visible on the fly body surface, whereas the bipolar neuron and sheath are located inside the body under the shaft. These components result from specialization of the four cells generated by successive divisions of the single Sensory Organ Precursor (SOP) cell.

About 500 cells of approximately 50 thousand cells of the imaginal disc enter neurogenesis during macrochaete formation [4]. The period of macrochaete development from the moment the SOP cells appear to the completion of its formation takes about 55 h during the late larval instar and early pupal instar [5-7].

The spatial location of SOP cells is identical to the bristle location on the imago body; correspondingly, the accuracy of bristle pattern depends on the correct SOP cell positioning [6, 7].

The sensor organ forms in three stages. Two of these stages are connected with the determinative point in the macrochaete morphogenesis–determination of SOP cell.

At the first stage, the so-called proneural clusters, groups of 20–30 cells, segregate from the massif of ectodermal cells of the wing imaginal disc. At the second stage, the SOP cell is determined and its position in the proneural cluster is specified, thereby determining the bristle position on the imago’s body. At the final stage, the SOP cell divides, and the daughter cells differentiate into the components of mechanoreceptor. Each stage has its own genetic control. 

Three gene groups are involved in the bristle morphogenesis, namely, proneural, which determine the segregation and location of proneural clusters; neurogenic, determining and positioning the SOP cell within the cluster; and selector, which specify the differentiation type for each daughter cell. The sequence of macrochaete formation stages is schematized in Fig. (**1**).

The critical factor predetermining the neural cell fate is the threshold level of proneural proteins, the products of *achaete–scute *(*AS-C*) gene complex. The control of this level is provided, on the one hand, by the intracellular regulation of *AS-C* activity and, on the other, by intercellular events mediated *via *the EGFR and Notch signaling pathways. This process involves dozens of genes.

Numerous papers have reported various aspects of functioning of the molecular genetic system involved in the control of macrochaete morphogenesis; however, its systematic description is yet absent. Here we analyze the available published data and propose an integral scheme for the functioning of the system controlling the macrochaete development in *D. melanogaster*.

## THE FIRST STAGE IN MACROCHAETE DEVELOPMENT: THE ROLE OF PRONEURAL GENES AND EGFR SIGNALING PATHWAY

The first stage in development of each bristle organ is formation of the proneural cluster, a group of cells with the neural fate that differentiate into cells of the peripheral nervous system. The proneural genes play the key role in this process. It is the expression of these genes that renders the cells of this cluster competent, i.e., able to become SOP cells [8].

Inactivation of proneural genes causes disappearance of some or all macrochaetes in imagoes. An ectopic expression of these genes due to switching of the ontogenetic mechanism from the epidermal to neural fate results in development of additional bristles at ectopic positions. Proneural genes can be divided into two classes, namely, (1) the genes of *achaete–scute *(*AS-C*) complex (*achaete, scute, lethal of scute,* and* asense*) and (2) the genes functionally and structurally close to the gene *atonal* (*atonal, amos,* and *cato*).

A correct functioning of the *AS-C* gene complex is essential for the bristle pattern formation. The constituent genes *achaete *(*ac*) and *scute *(*sc*) are the key in the hierarchy of genes involved in the bristle morphogenesis. Mutations in each of these genes appear as an allele-specific loss of individual bristles or their sets, while simultaneous inactivation of both genes leads to a complete disappearance of the bristles in adult fly [9, 10].

*AS-C* genes encode the bHLH family transcription factors, containing helix–loop–helix amino acid sequences and basic domains, though which they bind to the specific sites in the regulatory regions of the genes they control, E boxes [11]. Along with the proneural genes, *Delta, scabrous, E(spl)-C, charlatan, groucho, senseless,* etc., belong to such target genes.

*AS-C* occupies approximately 90 kbp in the genome and contains nine transcription units separated by untranscribed regions. The presence of the transcripts T5 (*ac*), T6 (*sc*), T3 (*lethal of scute, l’sc*), and T8 (T1a; *asense, ase*) play an important role for the macrochaete morphogenesis. Each transcript has its own time and spatial distribution profiles. The specificity and expression patterns of the *AS-C* genes are determined by enhancers, located at a distance of up to 100 kbp from this complex [12].

One type of enhancers initiates the expression of *ac* and *sc* genes in the cells of individual proneural cluster, and enhancers of the second type trigger this process in each SOP cell [13]. Activities of the enhancers of both types depend on a local combination of the transcription factors, or prepattern factors in the frame of Stern’s hypothesis [14, 15]. These factors are the products of both the proneural genes themselves and other genes, in particular, *u-shaped, pannier,* and *iroquois *complex genes (*arauca, caupolican,* and* mirror*) as well as some proteins of the EGFR signaling pathway [16-19].

For example, the *AS-C* complex in the mid-notum is activated by the protein Pannier, whereas in the lateral part, by the proteins encoded by the *iroquois* complex genes. In its turn, the expression of *pannier* and *iroquois *genes is regulated by the products of the genes *decapentaplegic* and *wingless,* respectively, of the EGFR signaling pathway cascade [18, 20-22].

Thus, the preciseness in bristle positioning is achieved through coordinated joint spatially limited expression of *AS-C* genes determined, on the one hand, by the prestructure—a set of the corresponding transcription factors—and, on the other, the system responding to the prestructure, containing the *AS-C* genes with their set of enhancers.

The cells of proneural cluster differ from the surrounding ectodermal cells in the content of AC–SC proteins: it is essentially higher in the cluster as compared with the neighbor ectodermal cells and reaches the maximal values in SOP cell. In addition, the SOP cells also accumulate the protein ASE. Several dozens of genes united by mutual and autoregulation involving the signaling pathways underlie this process.

### Expression Regulation of AS-C Genes

As *AS-C* proteins are transcription factors, they are able to regulate transcription, including transcription of the genes that code for them. These factors acquire a regulatory activity within heterodimers with certain other proteins. Depending on the composition, such complexes are either positive or negative regulators of *AS-C* gene expression.

The heterodimers of AC and SC with the protein DA, the product of gene *daughterless *(*da*), also a bHLH protein, are positive regulators of *AS-C* gene transcription. The transcription is activated through the binding of such heterodimers to the three E boxes in *AS-C* regulatory region [23].

The heterodimers of the proteins AS-C and EMC, the product of gene *extramacrochaete,* are negative regulators of the *AS-C* expression, as EMC is an HLH protein, deprived of the DNA-binding basic domain. The complexes formed by proneural proteins and EMC are incapable of binding to DNA. Competing with DA for binding to AS-C proteins, EMC decreases the concentration of functional heterodimers, thereby decreasing the transcription level of* AS-C* genes [24-27].

The activity of the proneural genes is regulated not only by the heterodimers containing AC and SC, but also by other factors.

Charlatan (CHN) is a direct transcription activator for the proneural genes. This transcription factor contains zinc finger domains and binds to the cluster-specific enhancers in *AS-C* regulatory region. An abnormal expression of gene *chn *leads to either loss of the macrochaetes (in the case of protein CHN deficiency) or development of extra macrochaetes (when surplus CHN is produced). In turn, the *chn* transcription in the cells of proneural clusters is activated by AS-C proteins [13].

The proteins produced by the neurogenic genes of the *Enhancer of split* (*E*(*spl*)*-C*) complex and *hairy *are direct negative activity regulators of the proneural genes.

*E*(*spl*)*-C* contains at least 11 transcription units [28]. It is assumed that transcription of the genes of this complex is activated with the involvement of proneural proteins [29]. Seven *E*(*spl*)*-C* transcripts encode the proteins of bHLH type carrying the WRPW tetrapeptides at their C end [30]. On the one hand, this structure makes them able to bind to DNA and, on the other, to form protein–protein complexes [28]. The target genes are repressed by a direct binding to their regulatory regions of both E(SPL)-С homo- and heterodimers and the E(SPL)-С proteins within the heterodimeric complexes with SC–DA [31].

In this process, the product of the neurogenic gene* groucho *(*gro*) is involved as a corepressor. GRO interacts with E(SPL)-С proteins with involvement of seven repeats in the highly conservative C-terminal domain WD4 (Trp–Arg–Pro–Trp) of GRO protein and the WRPW region of E(spl)-С proteins [19, 32, 33].

Hairy (H), a bHLH protein with the WRPW C-terminal region, is a direct repressor of *AS-C* gene transcription activity. This transcription factor binds to the C boxes (CACNNG) in the regulatory regions of the target genes [34]. In addition, H needs a cofactor, GRO, for its functional activity [32, 35, 36]. It is assumed that the H–GRO complex is involved in chromatin remodeling or interacts with the complex transcribing the target gene [37].

The transcription factor Senseless (SENS) plays a dual role in the activity regulation of proneural genes; this factor contains four zinc finger domains, through which it is capable of binding to both DNA and proneural proteins, direct activators of *sens* gene expression. SENS is an activator or a repressor of the proneural gene transcription depending on its content in the cell. At a low concentration, it acts as a repressor of the proneural gene activity, directly binding to DNA at the corresponding sites of *AS-C* enhancer regions; at a high concentration, it forms complexes with proneural proteins and DA, acting as a coactivator of the proneural gene expression. The activation is sensitive to the content of certain *E(spl)* proteins. It is assumed that the functional duality of SENS is connected with the conformational state of zinc finger domains and their different affinity for DNA and proneural proteins [38-41]. Thus, SENS acts as a switch of the proneural gene activity and, consequently, as a switch for the proneural cluster cell fate, thereby enhancing the SOP cell determination.

### EGFR Signaling Pathway and its Role in the Regulation of Drosophila Macrochaete Development

Along with a direct intracellular regulation of the proneural gene activity, the EGFR signaling pathway plays an important role in the macrochaete morphogenesis; this pathway brings about the so-called lateral cooperation. The genes constituting this signaling cascade are switched on at all the three stages of the sensor organ development, namely, formation of proneural clusters, SOP cell determination within this clusters, and cell specialization [19, 42].

Depending on the performed functions, the proteins of the EGFR signaling pathway can be divided into the following groups: (1) drosophila epidermal growth factor receptor, EGFR or DER; (2) its ligands, Spitz (SPI) and Argos (AOS); (3) the proteins involved in the ligand processing, Star (S) and Rhomboid (RHO); and (4) the proteins transducing signal from the cell surface into the nucleus (Ras/Raf/MAP kinase cascade and Pointed).

The transmembrane receptor DER belongs to the receptor tyrosine kinase family, the proteins with intrinsic kinase activity. The extracellular protein fragment comprises four domains; two of the domains, which provide the binding to ligand, are cysteine rich [43]. The receptor has two isoforms; however, the precise functions of these isoforms are yet vague [44].

The ligands for this receptor are SPI and AOS. An intracellular signal transduction is activated or blocked depending on the bound ligand [45-46].

Initially, SPI is synthesized as an inactive precursor and accumulated in the endoplasmic reticulum. Then the precursor is transferred by the protein Star to the Golgi apparatus, where it maturates. In the Golgi apparatus, the precursor–Star complex is cleaved by the RHO protease; then the mature ligand is transferred to the cell membrane [44, 47, 48].

The signal transduction commences from the SPI binding to the DER extracellular domain. Then the receptor’s intracellular domain in the recipient cell is phosphorylated and the Ras/Raf/MAP kinase cascade is activated. The intracellular signal transduction from the cell membrane to the nucleus initiates the transcription of gene *pointed *and the subsequent synthesis of two isoforms of the protein Pointed, Pnt-P1 and Pnt-P2 [49, 50]. The details of the intracellular signal transduction are still vague. Both isoforms can play the role of transcription factors for proneural genes, as they are capable of binding to the same DNA regions with their Est domains [51, 52]. The corresponding scheme is shown in Fig. (**2**).

The secreted ligand Argos is a repressor of the EGFR signaling pathway. The gene *argos* is activated simultaneously with the activation of proneural genes, and its expression is observed exclusively in the proneural cluster cells. Secretion of the ligand and its binding to the receptor blocks the EGFR signal transduction into the cells neighboring the cells actively expressing AS-C proteins [19, 54].

Thus, the local differential expression of *AS-C* genes and the EGFR signaling pathway determine a precise location of the proneural cluster and provide accumulation of proneural proteins in the cells of the cluster.

## THE SECOND STAGE OF MACROCHAETE DEVELOPMENT: NEUROGENIC GENES AND THE ROLE OF NOTCH SIGNALING PATHWAY

The second stage in the macrochaete formation comprises SOP determination and precise positioning within the proneural cluster and is controlled by a group of neurogenic genes. Abnormalities in their function make the majority of the cluster cells or even all cells neural. The obligatory condition for SOP cell determination is that the concentration of As-C proteins reaches the maximal value as compared with the neighboring cells. The cells with insufficient concentration of proneural proteins remain epidermal.

The lateral inhibition, which is mediated by the Notch signaling pathway leading to determination of only one cell as neural, is the master factor at this stage [30, 55, 56]. In the rest cells, the activity of proneural genes is repressed by a direct interaction of specific regulatory proteins of the Notch cascade with *AS-C* enhancer regions [57].

Several dozens of proteins, products of neurogenic genes, are involved in the Notch signaling cascade; the main proteins can be divided into the following groups:

- The gene encoding Notch receptor, *Notch* (*N*);
- The genes encoding Notch ligands *Delta* (*Dl*) and *Serrate* (*Ser*);
- The genes whose products provide the intercellular signal transduction, namely, *Presenilin* (*Ps*)*, kuzbanian* (*kuz*)*, polychaetoid* or* tamou *(*pyd* or* tam*)*, big brain *(*bib*)*, sanpodo *(*spdo*)*, *etc.;
- The genes whose products are involved in the receptor and ligand internalization, namely, *neuralized* (*neur*)*, Suppressor of deltex *(*Su*(*dx*))*, shibire,* *numb,* etc.; and
- The genes whose products provide the intercellular signal transduction, namely, genes of the *Enhancer of split* (*E*(*spl*)) and *Bearded* (*Brd-C*) complexes,* mastermind *(*mam*)*, Hairless *(*H*)*, Suppressor of Hairless *(*Su*(*H*))*,* *deltex *(*dx*), and several others.

The first three gene groups encode mainly transmembrane proteins and the proteins located on the cell surface and the other two, cytoplasmic and nuclear proteins.

The main components of the Notch signaling pathway are the Notch receptor, its ligand Delta, and the intracellular target—genes of the *Enhancer of split *(*E*(*spl*)*-C* complex. The products of these genes are the particular repressors of the proneural gene transcription.

Consider some components of the Notch signaling pathway in more detail.

### Notch Receptor

Notch receptor, the central element of this signaling pathway, is necessary for a correct development of the drosophila nervous system. Notch is a typical transmembrane protein comprising extra- and intracellular domains. The large extracellular domain contains 36 tandem conservative EGF-like repeats, involved in binding ligands, and three repeats of the cysteine-rich sequence N/LIN 12 [58]. The intracellular domain contains six tandem ankyrin repeats, the region containing 30 glutamine residues (opa repeat), and the PEST sequence, rich with proline, glutamine, serine, and threonine. It is assumed that the opa and PEST sequences are important for the regulation of protein stability [59].

Initially, N is synthesized as a protein with a molecular weight of about 300 kDa. It is then processed by proteases in the Golgi apparatus, and the mature receptor composed of intra- and extracellular domains appears on the cell surface [60, 61].

### Ligands of Notch Receptor

Delta is a transmembrane protein with a large extracellular domain containing nine EGF repeats and the conservative repeat DSL (Delta-Serrate-LAG-2) [30, 62].

The ligand Ser, functionally related to the protein DL, has the extracellular domain containing 14 EGF-like repeats, transmembrane domain, and a small intracellular part [63]. SER and DL are alternative ligands for the Notch receptor, as they interact with the same Notch extracellular fragment; however, the possibility of their intersubstitution is essentially limited [58, 64]. The glycosyltransferase Fringe, which inhibits the Notch ability to bind SER and enhances its binding to DL, selects the particular ligand [65, 66].

The interactions in the pair N–DL are the key interactions in the intercellular signal transduction within the proneural cluster, providing a correct progress of the process. It is known that the embryos homozygous at the mutations in *Dl* locus die as a result of nervous tissue hyperplasia. *Dl* expression is activated by the proneural proteins AC-SC. The accumulation of *Dl* protein in the future SOP cell and its binding to N receptors, localized on the membranes of neighboring cells, trigger the mechanism of lateral inhibition [55, 67].

### Intercellular Signal Transduction

This process is controlled by a large group of genes encoding the proteins mainly localized to the surface of the cell membrane or within it. The mechanisms of the action of *Presenilin, kuzbanian, polychaetoid *(*tamou*),* big brain*, and* sanpodo *genes are most well studied.

The genes *Ps, kuz, *and *pyd* encode the corresponding proteases, whose function in the Notch signaling pathway is in the cleaving of the mature N receptor into its extra- and intracellular domains [68-71].

The gene *bib* codes for a transmembrane domain belonging to the tunnel proteins and homologous to aquaporins, involved in the channel formation in the cytoplasmic membrane. It has been demonstrated that the mutants in gene *bib *contain a doubled number of sensor neurons, i.e., similar to the other neurogenic genes, its role is in the determination of fate for the proneural cluster cells. It has been also shown that BIB protein is necessary for receiving the lateral inhibition signal or responding to it rather than for generating such signal. The BIB action is synergistic with the DL and N and, presumably, enhances their binding or is involved in the next stage of signal transduction; however, the precise mechanism is yet unknown [72, 73].

The gene *spdo* encodes a transmembrane protein, an activator of the Notch signaling pathway. The mutants in this gene develop two neurons instead of the neuron and the glial cell [74]. According to one of the hypotheses, formation of the N–SPDO complex allows the protease PS to correctly cleave the N receptor [75]. According to another opinion, the function of SPDO protein is in the regulation of N endocytosis [76].

Some of the neurogenic genes encode the proteins that are not only involved in the Notch signaling pathway, but also directly influence the expression of regulator genes for proneural gene activity. In particular, *pyd* inhibits the proneural gene expression being a direct activator of *extramacrochaete* gene transcription [68].

### Internalization of Receptor and Ligands

The ubiquitin ligases Neuralized (NEUR) and Suppressor of deltex (Su(DX)) are involved in this process as well as the proteins Dynamin (DYN) и Numb, which function as activators or inhibitors of this signaling pathway.

NEUR and DYN maintain an inductive state of the signal-sending cell and are positive regulators of the Notch cascade. Attaching ubiquitin molecules to the complex of the ligand DL with the Notch extracellular domain, Neur initiates its transport to the inducer cell [77-79].

The internalization of the complex DL–N extracellular domain into the inducer cell depends also on the protein DYN, coded for by the gene *shibire*. DYN displays a GTPase activity and cleaves the vesicle with the transported proteins from the membrane, thus vacating the place for new ligand molecules on the membrane of inducer cell. This allows for formation of new ligand–receptor complexes, thereby prolonging the cell inductive state [78, 79]. It has been demonstrated that the internalization of this complex influences the intracellular signal transduction in the recipient cell as well; however, the mechanism of this effect is still unknown [78, 80].

The ubiquitin ligase Su(DX) and protein Numb are negative regulators for the Notch cascade, acting *via *the internalization and transformation of the N receptor in the recipient cell.

Su(DX) assists the full-sized receptor in entering the cell. In the cell, the complex Su(DX)–N in the late endosome triggers the receptor degradation mechanisms. Thus, the receptor outflow from the cell membrane is provided [78, 79].

The protein Numb interrupts the Notch signal transduction and blocks the overall signaling pathway. It has been demonstrated that this effect is connected with the inactivation of N receptor caused by its direct interaction with Numb [81]. Recent data demonstrate that Numb induces endocytosis of the full-sized receptor by the recipient cell. The adapter protein α-Adaptin and, presumably, SPDO, forming the complex with receptor, are involved in this process [75, 79, 82-84].

### Intracellular Signal Transduction

Intracellular signal transduction is provided by the genes of *Enhancer of split *and* Bearded* complexes, *mastermind*,* Suppressor of Hairless, Hairless, deltex*, and several other genes.

The *E*(*spl*) complex genes are the intracellular target and the final element of the Notch cascade. In neurogenesis, *E*(*spl*)*-C* is an antagonist of the proneural genes as activators of the neural pathway of cell development. The proteins E(spl)-С act as repressors, inhibiting the transcription of proneural genes. It has been demonstrated that the role of *E(spl)-C* proteins in the neurogenesis consists in repression of not only the proneural genes, but also their target genes, in particular, *deadpan, neuralized, scabrous,* etc. Moreover, an indispensable condition of *E(spl)-C* protein activities is the presence of the cofactor GRO [29, 33].

The *Bearded* complex comprises six genes encoding small proteins not belonging to bHLH type and carrying an α-helix at their N termini [85, 86]. It is assumed that the BRD family proteins are involved in the regulation of Notch cascade through influencing DL endocytosis [87]. Su(H) and proneural proteins activate *Brd-C* expression [88, 89].

Another neurogenic gene, *mastermind*, codes for the nuclear protein MAM, composed of alternating acidic and basic domains, suggesting its DNA binding ability [90]. In the complex with Su(H), formed by MAM only in the presence of Notch intracellular domain, this protein acts as a strong transcription coactivator for the target genes of the Notch signaling pathway, in particular, *E*(*spl*)*-C* genes [91-93]. The antagonistically acting genes *Suppressor of Hairless* (*Su*(*H*)) and *Hairless *(*H*) play an exclusively important role in the Notch signaling pathway [94]. The mutants in gene *H* display an abnormal determination of SOP cells and, as a consequence, the absence of bristles. *Su*(*H*) is a dominant suppressor of the phenotype *H.* An elevated expression of these genes has the same phenotypic manifestation, namely, appearance of additional bristles.

The protein Su(H) is among the key elements in the Notch signaling pathway, as it is involved in the signal transduction from the cell membrane to the nucleus and is a direct activator of *E*(*spl*)*-C* transcription.

The signal is transduced by the complex Su(H)–N intracellular domain, which is formed when Su(H) binds to the ankyrin repeats of the receptor [95]. After this complex is transported to the nucleus and binds the protein MAM, Su(H) within the complex specifically binds to the consensus sequence 5'-GTGRGAR-3' in the regulatory regions of *E*(*spl*)*-C* genes, thereby initiating their transcription [96]. It is assumed that the binding specificity is provided by the Su(H) integrase domain [97].

The basic protein Hairy is an antagonist of Su(H) as an activator of the *E*(*spl*)*-C* gene transcription. The complex comprising H, Su(H) and the corepressors dCtBP (Drosophila C-terminal binding protein) and Gro is the functionally active repressor of its target genes [93]. H interacts with the corepressor dCtBP *via *the C-terminal sequence PLNLS and with GRO, *via *the sequence YSIHSLLG (the so-called eh1 motif) [98-100]. Both corepressors attract histone deacetylase into the complex, thereby decreasing the level of chromatin acetylation in the corresponding regions and, as a consequence, decrease in their transcription activity. However, it is still unclear whether the interactions of both corepressors with the complex are mutually exclusive or these corepressors act at fundamentally different levels of the target gene repression [101].

An important function of the protein GRO is in the switching of activities of the signaling pathways acting in different directions. It has been demonstrated that the GRO phosphorylation by MAPK (EGFR signaling pathway) weakens the GRO-dependent repression of *E*(*spl*)*-С* (Notch signaling pathway) [102-103]. GRO is a corepressor for many regulatory molecules; therefore, a change in its activity can influence a wide range of genes in the expression regulation of which GRO is involved. In the macrochaete morphogenesis, GRO plays a dual role, namely, (1) Gro in the complex with Su(H)-H inhibits *E*(*spl*)*-C* gene activities in the cells with SOP fate and (2) in the complex with *E(spl)-С* proteins, represses the transcription of *AS-C* genes in the cells surrounding the proneural cluster. In the former case, the content of proneural proteins in the cells of the cluster increases and in the latter, decreases [92, 100].

Deltex, the product of gene *deltex*, is a basic protein containing three domains separated by the sequence regions rich with glutamine. A zinc finger domain is located at its C terminus; this domain provides the interaction of Deltex with other proteins, in particular, binding to the N intracellular domain in the region of ankyrin repeats [104-106]. It is assumed that the interaction between N and DX enhances an accelerated transport of the complex Su(H)–N intracellular domain into the recipient cell nucleus [106]. It has been recently demonstrated that DX can stabilize the receptor, preventing its degradation in lysosomes, thereby contributing to the retention of the pool of functional full-value receptor molecules [79, 107]. Thus, DX acts as a positive regulator with the Notch signaling pathway.

The data briefed above suggest the scheme describing the Notch signaling pathway functioning shown in Fig. (**3**).

Since all the cell of ectodermal proneural cluster express the proneural proteins AS-C, receptor N, and ligand DL, each cell has either neural or ectodermal fate potential and can be either signal-transmitting or signal-receiving. Random fluctuations in the contents of these proteins in cells are increased *via *the feedback cycles; consequently, proneural proteins reach an suprathreshold concentration in one of the cells, thereby activating the DL synthesis. Later, this cell will become the SOP cell. In the other cells, the lateral inhibition, mediated by the Notch signaling pathway, is triggered.

The Notch signaling pathway is activated by the binding between the extracellular domains of receptor N, localized to the surface of signal-receiving cell, and the ligand DL, localized to the membrane of signal-inducing cell. The receptor–ligand interaction takes place in the intercellular space between two adjacent proneural cluster cells [65, 66].

In the recipient cell, the proteases KUZ and PS cleave the N intercellular domain [70, 71, 78, 108]. Then the intercellular domain within the complex with Su(H) is transported to the nucleus, where MAM is attached to this complex. The signal transduction *via *the Notch pathway ends by *E*(*spl*)*-C* transcription activation upon a site-specific binding of Su(H) to its regulatory regions. The proteins E(SPL)-C inhibit transcription of its target genes, first and foremost, the proneural genes, and deprive the recipient cell of a neural fate potential [91, 109].

The complex between the receptor’s extracellular domain and ligand is transported into the inducer cell, i.e., future SOP cell, to be completely degraded. Dynamin and NEUR proteins are involved in internalization of the complex [78, 80, 110]. An unidirectional transduction of the Notch signal prohibits the synthesis of *E(spl)-C* proteins in the inducer cell, whereas the synthesis of proneural proteins is continued in it to the level providing its SOP cell fate.

Thus, the functioning of the Notch signaling pathway determines the single cell of the proneural cluster with a neural fate, whereas the rest cells will remain epidermal.

The lateral inhibition is efficient for the cells directly adjacent to the presumptive SOP cell. However, the neurogenic gene *scabrous *(*sca*) acquires an exclusive role in determining the fate of more remote cells of the proneural cluster; expression of this gene is activated by the heterodimers of AS-C and DA proteins [111, 112].

SCA is a secreted protein, carrying a sequence similar to fibrinogen β- and γ-chains at its carboxyl end [111, 113]. It has been found that SCA is necessary for the determination and maintenance of the adhesive characteristics of ectodermal cells. SCA is capable of binding to N yet is not its active ligand. In its absence, the neural developmental pathway is not blocked in the cells that do not directly contact the future SOP cell. On the other hand, SCA is not necessary for the lateral inhibition of the cells contacting the SOP cell, as Dl is sufficient for this process. Since SCA distribution gradient is observed within the proneural cluster, it is assumed that this is the particular factor that determined the size of the region where the inhibiting signal is spread. However, the precise mechanism of SCA action in the lateral inhibition process is still vague. Presumably, this protein is necessary for stabilization of the N–DL complex [112].

## THE THIRD STAGE OF MACROCHAETE DEVELOPMENT: THE ROLE OF SELECTOR GENES

The lateral inhibition ends by determination of the single proneural cluster cell as a SOP cell. Then the determined cell undergoes two successive divisions, which give four specialized cells, namely, trichogen, tormogen, nerve cell, and thecogen, which then develop into individual components of the bristle organ: shaft; socket, surrounding its base; bipolar neuron; and glial cell. The main mechanism underlying the cell diversity is an asymmetric cell division, which makes the daughter cell different from the parental cell and from one another in the ability to differentiate in a particular direction.

This process is controlled by the selector genes* tramtrack* (*ttk*), *musashi* (*msi*), and *prospero* (*pros*). At this stage, two neurogenic genes, *numb* and *neuralized,* play the role of selectors.

The genes *numb* and* neur* encode membrane proteins. The role of proteins Numb and NEUR in the daughter cell specialization is determined by their asymmetric location in the SOP cell—they are localized to only one of its poles. Thus, the distribution of Numb and NEUR between the daughter cells is different already upon the first mitotic division, as these proteins segregate into one cell [79, 114, 115]. As a consequence of this asymmetric division, the daughter cells also differ in both the contents of other proteins involved in the macrochaete morphogenesis, including regulatory proteins, and the modulation of their target gene activities.

The daughter cell that received Numb and NEUR follows the neural specialization to form neuron and thecogen, as Numb blocks the transmission of Notch signal into this cell, while NEUR enhances the signal transduction into the other cell free of the proteins in question. The absence of Numb and NEUR in the second daughter cell determines the ability of this cell to adequately receive the Notch signal, which blocks the neural fate; thus, this cell gives rise to the trichogen and tormogen [79, 81].

The gene *ttk *codes for a nuclear protein; its mutations lead to the development of additional neurons at the expense of the other bristle organ components. The protein TTK appears in one of the two daughter cells produced by the first division of SOP cell. In the next division, this particular cell gives rise to the trichogen and tormogen. It has been demonstrated that TTK appearance there is determined by activation of the Notch pathway; however, the mechanism of this correlation is unclear [116, 117].

Although the protein TTK is undetectable in the second daughter cell, the contents of *ttk* mRNA in both cells are approximately equal. It has been demonstrated that the differences in TTK content are connected with the action of another selector gene, *musashi *[116, 117].

The gene *msi* encodes a nuclear protein expressed at all the stages of mechanoreceptor development and able to prevent the translation of *ttk* mRNA by binding specifically to its 3’-untranslated region. Mutations in *msi *gene lead to formation of additional glial cell instead of neuron [116, 118, 119]. The protein MSI is detectable in both daughter cells after the first SOP cell division; in this process, this protein prohibits the translation of *ttk *transcript only in one of them, the cell that gives the neural components of the bristle organ—neuron and thecogen. This is the cell that have received the Numb protein as a result of the asymmetric division and where the Notch signal is blocked. In the other daughter cell, where the Notch signal transduction is not blocked, the MSI activity is inhibited and *ttk* mRNA is translated. This cell further gives rise to trichogen and tormogen [116].

The gene* pros *codes for a transcription factor, which carries a homeodomain and conserved Prospero domain (Pros domain), localized to the carboxyl end of the molecule [120]. At present, both these domains are regarded as a single homeo–Pros domain, necessary for binding to the specific DNA sites [121, 122]. The protein PROS is detectable both in the nucleus and cytoplasm, and its distribution between the nucleus and cytoplasm is a dynamic process. It has been demonstrated that the homeo–Pros domain is responsible for withdrawing the protein from the nucleus; however, this process requires further studies [123].

PROS determines the neural fate of the SOP cell derivatives. This protein is first detectable in the nucleus of only one of the SOP daughter cells, the particular cell that forms after division the neural components of the bristle organ. During mitosis, PROS is transferred to the membrane, where it is located together with Numb. Then PROS appears in the neuron and thecogen. The dynamics of changes in the PROS contents in these cells are diametrically opposite: it is decreasing in the neuron and increasing in the thecogen. The PROS protein is never detectable in the SOP cell, its derivatives from the first division, which then differentiate into tormogen and trichogen, as well as in the tormogen and trichogen themselves [124].

Thus, the cell asymmetrical division and selector gene activities determine the further fate of SOP daughter cells as different components of the sensor organ.

## CONCLUSION

Formation of the full-fledged bristle pattern on drosophila body is a result of successive limitation of the potencies of ectodermal cells in the imaginal disc.

The macrochaete development is controlled *via *the system of dynamic intra- and intercellular processes. Functioning of this system is provided by a wide network of genes interconnected with the mechanisms of cross- and autoregulation, which underlie a fine tuning of their activities. A correct functioning of this system guarantees the formation of a full-fledged bristle pattern, i.e., a fixed number of macrochaetes at stringently determined positions.

**Fig. (4):**
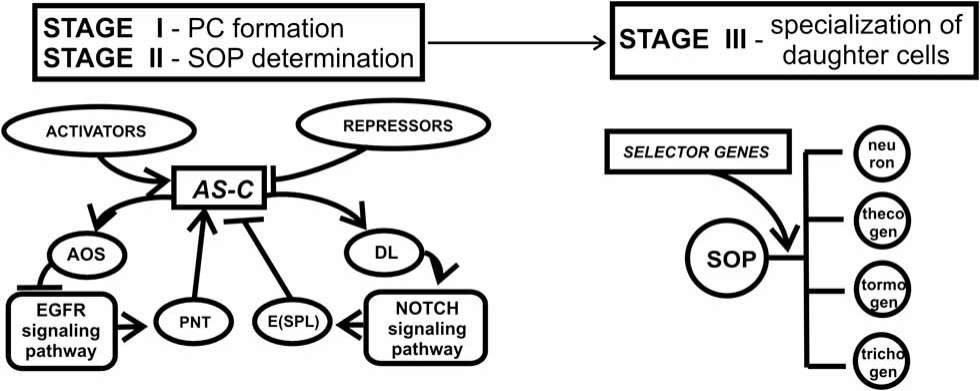
Scheme of the system controlling macrochaete development in *D. melanogaster*: PC, proneural cluster; SOP, sensor organ precursor cell; *AS-C,* genes of *achaete–scute* complex; Pnt, Pointed; E(spl), proteins of *Enhancer of split* complex; Aos, Argos; and Dl, Delta. Arrows indicate activation events and lines with stub ends, repressor events.

Analysis of the relevant published data suggests the following integrated scheme for the control of these three stages in macrochaete development (Fig. **4**).

The genes of the *achaete–scute* complex play the key yet dual role in the regulation of macrochaete development. First, they initiate the bristle development providing its first stage—definition of the proneural cell clusters. The competence of cells within the cluster is determined by a certain threshold content of the proneural proteins, which is created and maintained through the expression control of the genes of this complex *via *the EGFR signaling pathway, their autoregulation by AS–C/DA heterodimers, and *trans-*regulation *via *the interactions with positive (SENSE, CHA, and PNT) and negative (H and EMC) regulators of its transcriptional activity.

Second, *AS-C* genes are involved in the determination of SOP cell within proneural cluster. A cell determined as SOP cell should contain the AS-C proneural proteins at a concentration exceeding the threshold level. Being transcription factors, proneural proteins activate expression of *Delta,* triggering the gene cascade of the Notch signaling pathway; the final event in this pathway is the expression inhibition of *AS-C* genes and/or their target genes by the E(SPL)-C repressor proteins in all the proneural cluster cells except for SOP cell, where the content of proneural proteins reaches the required suprathreshold values.

Thus, *AS-C* closes the circuit of activation–inhibition interactions in the chain of proneural and neurogenic genes, which determine the conditions of local expression of this complex in the cells of imaginal disc ectodermal layer and lead to determination of the SOP cells.

An asymmetric division of the SOP cell and daughter cell specialization is controlled by selector genes.

Drosophila macrochaete is used as a model system for studying the mechanisms of cell specialization already for over 50 years. During this time, the general molecular genetic structure of *AS-C* region and its role in the macrochaete morphogenesis have been clarified as well as the expression pattern of proneural gene expression; certain specific details of the prepattern signals, which are identified as the transcription factors interacting with *AS-C* complex enhancers have been found out; the signaling pathways and genes providing the signal transduction *via *these pathways have been found; a deeper insight into the mechanisms of lateral cooperation, lateral inhibition, and asymmetric division has been reached; the list of genes involved in the macrochaete morphogenesis has been considerably supplemented; and the functions of many known players in this process have been detailed.

It has been found out quite recently that gene expression is also regulated at the level of posttranscriptional silencing with involvement of miRNA; this mechanism can specifically block the translation of certain mRNA targets and additionally control production of the corresponding proteins in the cell. Over five hundred genes whose expression can be controlled by the posttranslational silencing have been theoretically predicted for drosophila. This list includes also the genes involved in macrochaete morphogenesis—*neur, dx, fng*, *E(spl)-C), hairy,* *sens, *and* Bearded* [125, 126]. The regulation throughout the RNA interference has been shown experimentally for *hairy*, *E(spl)* and *sens *[127, 128].

Nonetheless, despite an essential success in understanding the processes involved in the development of drosophila peripheral nervous system, neither the complete list of the genes involved in the bristle pattern formation programs nor the mechanisms implementing these programs have been determined. The precise time parameters and quantitative characteristics for the overwhelming majority of these processes are yet unknown.

This review briefs only the main elements and events of the multidimensional process of drosophila macrochaete development. A tremendous volume of experimental data has been so far accumulated, and their comprehensive analysis requires the state-of-the-art methods of bioinformatics, which make it possible to correctly describe, formalize, and simulate the formation of both individual macrochaetes and overall bristle pattern.

## Figures and Tables

**Fig. (1):**
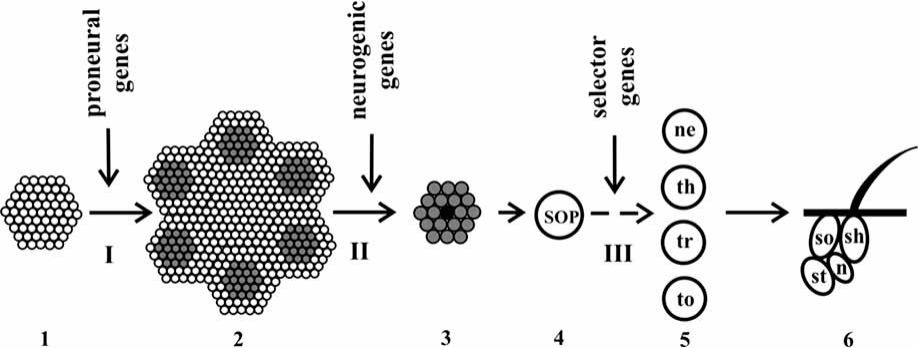
Scheme of development of *D. melanogaster* mechanoreceptors. I, II, and III are the stages of mechanoreceptor development (see text for details); (1) ectodermal cells of the wing imaginal disc; (2) proneural clusters (gray) in the wing imaginal disc; (3) proneural cluster with SOP cell (in the center, shown black); (4) SOP cell; (5) daughter cells of SOP cell (ne, neuron; th, thecogen; tr, trichogen; and to, tormogen); and (6) bristle organ (n, bipolar neuron; st, sheath; sh, shaft; and so, socket).

**Fig. (2):**
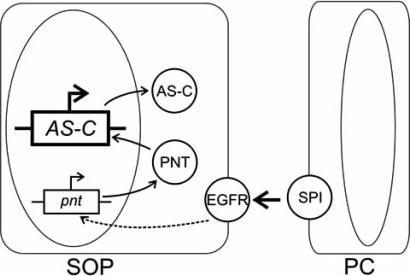
Scheme of involvement of the EGFR signaling pathway in the activation of *AS-C* genes [53]. Ovals denote cell nuclei; gene names are italicized; protein names are given in Roman type; AS-C, achaete–scute complex; pnt, pointed; spi, spitz; EGFR, epidermal growth factor receptor; SOP, sensor organ precursor cell; PC, proneural cluster cell; and arrows indicate activation events.

**Fig. (3):**
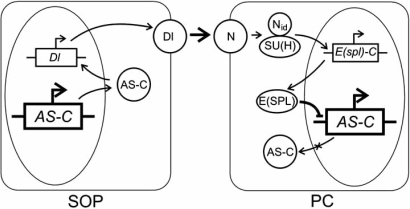
Scheme of involvement of the Notch signaling pathway in the regulation of transcription activity of *AS-C* genes [53]. SOP is sensor organ precursor cell; PC, proneural cluster cell; protein names are given in Roman type; gene names, in italics; AS-C, achaete–scute complex; Dl, Delta; N, Notch; E(spl), Enhancer of split; Nid, Notch intracellular domain; su(H), suppressor of Hairless. Arrows indicate activation events and lines with stub ends, repressor events.

## References

[1] Usui K, Pistillo D, Simpson P (2004). Mutual exclusion of sensory bristles and tendons on the notum of dipteran flies. Curr. Biol.

[2] Simpson P, Marcellini S (2006). The origin and evolution of stereotyped patterns of macrochaetes on the nota of cyclorraphous *Diptera*. Heredity.

[3] García-Bellido A, Lloyd CW, Rees DA (1981). From the gene to the pattern: chaeta differentiation. Cellular controls in differentiation.

[4] García-Bellido A, Merriam JR (1971). Parameters of the Wing Imaginal Disc Development of *Drosophila melanogaster*. Dev. Biol.

[5] Hartenstein V, Posakony JW (1989). Development of adult sensilla on the wing and notum of Drosophila melanogaster. Development.

[6] Huang F, Dambly-Chaudière C, Ghysen A (1991). The emergence of sense organs in the wing disc of Drosophila. Development.

[7] Campuzano S, Modolell J (1992). Patterning of the *Drosophila* nervous system: the *achaete-scute* gene complex. Trends Genet.

[8] Reeves N, Posakony JW (2005). Genetic programs activated by proneural proteins in the developing *Drosophila* PNS. Dev. Cell.

[9] Dubinin NP (1964). The theory of gene: history and modern problems. Bull. Moscow Soc. Naturalists (Biological Series).

[10] Ghysen A, Dambly-Chaudiere C (1988). From DNA to form: the achaete-scute complex. Genes Dev.

[11] Powell LM, Zur Lage PI, Prentice DR, Senthinathan B, Jarman AP (2004). The proneural proteins Atonal and Scute regulate neural target genes through different E-box binding sites. Mol. Cell Biol.

[12] Gomez-Skarmeta JL, Rodriguez I, Martinez C, Culi J, Ferres-Marco D, Beamonte D, Modolell J (1995). Cis-regulation of *achaete* and *scute*: shared enhancer-like elements drive their coexpression in proneural clusters of the imaginal discs. Genes Dev.

[13] Escudero LM, Caminero E, Schulze KL, Bellen HJ, Modolell J (2005). Charlatan, a Zn-finger transcription factor, establishes a novel level of regulation of the proneural *achaete/scute* genes of *Drosophila*. Development.

[14] Stern C (1954). Two or three bristles. Am. Sci.

[15] Gómez-Skarmeta JL, Campuzano S, Modolell J (2003). Half a century of neural prepatterning: the story of a few bristles and many genes. Nat. Rev. Neurosci.

[16] Leyns L, Gómez-Skarmeta JL, Dambly-Chaudiere C (1996). *iroquois*: a prepattern gene that controls the formation of bristles on the thorax of *Drosophila*. Mech. Dev.

[17] Haenlin M, Cubadda Y, Blondeau F, Heitzler P, Lutz Y, Simpson P, Ramain P (1997). Transcriptional activity of *pannier* is regulated negatively by heterodimerization of the GATA DNA-binding domain with a cofactor encoded by the *u-shaped* gene of *Drosophila*. Genes Dev.

[18] Garcia-Garcia MJ, Ramain P, Simpson P, Modolell J (1999). Different contributions of *pannier* and *wingless* to the patterning of the dorsal mesothorax of *Drosophila*. Development.

[19] Culi J, Martin-Blanco E, Modolell J (2001). The EGF receptor and N signalling pathways act antagonistically in *Drosophila* mesothorax bristle patterning. Development.

[20] Tomoyasu Y, Nakamura M, Ueno N (1998). Role of dpp signalling in prepattern formation of the dorsocentral mechanosensory organ in *Drosophila melanogaste*. Development.

[21] Phillips RG, Warner NL, Whittle JR (1999). Wingless signaling leads to an asymmetric response to decapentaplegic-dependent signaling during sense organ patterning on the notum of *Drosophila melanogaster*. Dev. Biol.

[22] Calleja M, Renaud O, Usui K, Pistillo D, Morata G, Simpson P (2002). How to pattern an epithelium: lessons from *achaete-scute* regulation on the notum of *Drosophila*. Gene.

[23] Cabrera CV, Alonso MC (1991). Transcriptional activation by heterodimers of the *achaete-scute* and *daughterless* gene products of *Drosophila*. EMBO J.

[24] Van Doren M, Powell PA, Pasternak D, Singson A, Posakony JW (1992). Spatial regulation of proneural gene activity: auto- and cross-activation of *achaete* is antagonized by *extramacrochaetae*. Genes Dev.

[25] Vaessin H, Brand M, Jan LY, Jan YN (1994). *daughterless* is essential for neuronal precursor differentiation but not for initiation of neuronal precursor formation in *Drosophila* embryo. Development.

[26] Cabrera CV, Alonso MC, Huikeshoven H (1994). Regulation of *scute* function by *extramacrochaete in vitro* and *in vivo*. Development.

[27] Smith JE 3rd, Cronmiller C (2001). The *Drosophila daughterless* gene autoregulates and is controlled by both positive and negative cis regulation. Development.

[28] Giebel B, Campos-Ortega JA (1997). Functional dissection of the *Drosophila* Enhancer of split protein, a suppressor of neurogenesis. Proc. Natl. Acad. Sci. USA.

[29] Giagtzoglou N, Koumbanakis KA, Fullard J, Zarifi I, Delidakis C (2005). Role of the Sc C terminus in transcriptional activation and E(spl) repressor recruitment. J. Biol. Chem.

[30] Artavanis-Tsakonas S, Matsuno K, Fortini ME (1995). Notch signaling. Science.

[31] Heitzler P, Bourouis M, Ruel L, Carteret C, Simpson P (1996). Genes of the *Enhancer of split* and *achaete-scute* complexes are required for a regulatory loop between Notch and Delta during lateral signalling in *Drosophila*. Development.

[32] Paroush Z, Finley RL Jr, Kidd T, Wainwright SM, Ingham PW, Brent R, Ish-Horowicz D (1994). Groucho is required for *Drosophila* neurogenesis, segmentation, and sex determination and interacts directly with hairy-related bHLH proteins. Cell.

[33] Giagtzoglou N, Alifragis P, Koumbanakis KA, Delidakis C (2003). Two modes of recruitment of E(spl) repressors onto target genes. Development.

[34] Van Doren M, Bailey AM, Esnayra J, Ede K, Posakony JW (1994). Negative regulation of proneural gene activity: hairy is a direct transcriptional repressor of *achaete*. Genes Dev.

[35] Fisher AL, Caudy M (1998). The function of hairy-related bHLH repressor proteins in cell fate decisions. BioEssays.

[36] Bianchi-Frias D, Orian A, Delrow JJ, Vazquez J, Rosales-Nieves AE, Parkhurst SM (2004). Hairy transcriptional repression targets and cofactor recruitment in *Drosophila*. PLoS Biol.

[37] Courey AJ, Jia S (2001). Transcriptional repression: the long and the short of it. Genes Dev.

[38] Nolo R, Abbott LA, Bellen HJ (2000). Senseless, a Zn finger transcription factor, is necessary and sufficient for sensory organ development in *Drosophila*. Cell.

[39] Jafar-Nejad H, Acar M, Nolo R, Lacin H, Pan H, Parkhurst SM, Bellen HJ (2003). Senseless acts as a binary switch during sensory organ precursor selection. Genes Dev.

[40] Jafar-Nejad H, Tien AC, Acar M, Bellen HJ (2006). Senseless and Daughterless confer neuronal identity to epithelial cells in the *Drosophila* wing margin. Development.

[41] Acar M, Jafar-Nejad H, Giagtzoglou N, Yallampalli S, David G, He Y, Delidakis C, Bellen HJ (2006). Senseless physically interacts with proneural proteins and functions as a transcriptional coactivator. Development.

[42] Freeman M (1998). Complexity of EGF receptor signalling revealed in *Drosophila*. Curr. Opin. Genet. Dev.

[43] Livneh E, Glazer L, Segal D, Schlessinger J, Shilo BZ (1985). The *Drosophila EGF receptor* gene homolog: conservation of both hormone binding and kinase domains. Cell.

[44] Shilo BZ (2003). Signaling by the *Drosophila* epidermal growth factor receptor pathway during development. Exp. Cell Res.

[45] del Alamo D, Terriente J, Diaz-Benjumea FJ (2002). Spitz/EGFr signalling *via* the Ras/MAPK pathway mediates the induction of bract cells in *Drosophila* legs. Development.

[46] Klein DE, Nappi VM, Reeves GT, Shvartsman SY, Lemmon MA (2004). Argos inhibits epidermal growth factor receptor signalling by ligand sequestration. Nature.

[47] Tsruya R, Schlesinger A, Reich A, Gabay L, Sapir A, Shilo BZ (2002). Intracellular trafficking by Star regulates cleavage of the *Drosophila* EGF receptor ligand Spitz. Genes Dev.

[48] Urban S (2006). Rhomboid proteins: conserved membrane proteases with divergent biological functions. Genes Dev.

[49] Gabay L, Seger R, Shilo BZ (1997). *In situ* activation pattern of *Drosophila* EGF receptor pathway during developmenmt. Science.

[50] Kumar JP, Hsiung F, Powers MA, Moses K (2003). Nuclear translocation of activated MAP kinase is developmentally regulated in the developing *Drosophila* eye. Development.

[51] Albagli O, Klaes A, Ferreira E, Leprince D, Klambt C (1996). Function of ets genes is conserved between vertebrates and *Drosophila*. Mech. Dev.

[52] zur Lage PI, Powell LM, Prentice DR, McLaughlin P, Jarman AP (2004). EGF receptor signaling triggers recruitment of *Drosophila* sense organ precursors by stimulating proneural gene autoregulation. Dev. Cell.

[53] Bukharina TA, Katokhin AV, Furman DP (2006). The gene network determining development of *Drosophila melanogaster* mechanoreceptors. Proc. Fifth Int. Conf. BGRS.

[54] Golembo M, Schweitzer R, Freeman M, Shilo BZ (1996). *Argos* transcription is induced by the *Drosophila* EGF receptor pathway to form an inhibitory feedback loop. Development.

[55] Heitzler P, Simpson P (1991). The choice of cell fate in the epidermis of *Drosophila*. Cell.

[56] Ghysen A, Thomas R (2003). The formation of sense organs in *Drosophila*: a logical approach. BioEssays.

[57] Culi J, Modolell J (1998). Proneural gene self-stimulation in neural precursors: an essential mechanism for sense organ development that is regulated by Notch signaling. Genes Dev.

[58] Fortini ME, Artavanis-Tsakonas S (1993). Notch: neurogenesis is only part of the picture. Cell.

[59] Wharton KA, Johansen KM, Xu T, Artavanis-Tsakonas S (1985). Nucleotide sequence from the neurogenic locus *Notch* implies a gene product that shares homology with proteins containing EGF-like repeats. Cell.

[60] Rand MD, Grimm LM, Artavanis-Tsakonas S, Patriub V, Blacklow SC, Sklar J, Aster JC (2000). Calcium depletion dissociates and activates heterodimeric Notch receptors. Mol. Cell Biol.

[61] Kopan R (2002). Notch: a membrane-bound transcription factor. J. Cell Sci.

[62] Sun X, Artavanis-Tsakonas S (1997). Secreted forms of DELTA and SERRATE define antagonists of Notch signaling in *Drosophila*. Development.

[63] Fleming RJ, Scottgale TN, Diederich RJ, Artavanis-Tsakonas S (1990). The gene *Serrate* encodes a putative EGF-like transmembrane protein essential for proper ectodermal development in *Drosophila melanogaster*. Genes Dev.

[64] Gu Y, Hukriede NA, Fleming RJ (1995). *Serrate* expression can functionally replace *Delta* activity during neuroblast segregation in the *Drosophila* embryo. Development.

[65] Panin VM, Papayannopoulos V, Wilson R, Irvine KD (1997). Fringe modulates Notch-ligand interactions. Nature.

[66] Schweisguth F (2004). Regulation of Notch signaling activity. Curr. Biol.

[67] Kunisch M, Haenlin M, Campos-Ortega JA (1994). Lateral inhibition mediated by the *Drosophila* neurogenic gene *delta* is enhanced by proneural proteins. Proc. Natl. Acad. Sci. USA.

[68] Chen CM, Freedman JA, Bettler DR Jr, Manning SD, Giep SN, Steiner J, Ellis HM (1996). Polychaetoid is required to restrict segregation of sensory organ precursors from proneural clusters in *Drosophila*. Mech. Dev.

[69] Guo Y, Livne-Bar I, Zhou L, Boulianne GL (1999). *Drosophila presenilin* is required for neuronal differentiation and affects notch subcellular localization and signaling. J. Neurosci.

[70] Struhl G, Greenwald I (2001). Presenilin-mediated transmembrane cleavage is required for Notch signal transduction in *Drosophila*. Proc. Natl. Acad. Sci. USA.

[71] Lieber T, Kidd S, Young MW (2002). Kuzbanian-mediated cleavage of *Drosophila* Notch. Genes Dev.

[72] Rao Y, Bodmer R, Jan LY, Jan YN (1992). The *big brain* gene of *Drosophila* functions to control the number of neuronal precursors in the peripheral nervous system. Development.

[73] Doherty D, Jan LY, Jan YN (1997). The *Drosophila* neurogenic gene *big brain*, which encodes a membrane-associated protein, acts cell autonomously and can act synergistically with *Notch* and *Delta*. Development.

[74] Dye CA, Lee JK, Atkinson RC, Brewster R, Han PL, Bellen HJ (1998). The *Drosophila sanpodo* gene controls sibling cell fate and encodes a tropomodulin homolog, an actin/tropomyosin-associated protein. Development.

[75] O'Connor-Giles KM, Skeath JB (2003). Numb inhibits membrane localization of Sanpodo, a four-pass transmembrane protein, to promote asymmetric divisions in *Drosophila*. Dev. Cell.

[76] Hutterer A, Knoblich JA (2005). Numb and alpha-Adaptin regulate Sanpodo endocytosis to specify cell fate in *Drosophila* external sensory organs. EMBO Rep.

[77] Lai EC, Deblandre GA, Kintner C, Rubin GM (2001). *Drosophila* Neuralized is a ubiquitin ligase that promotes the internalization and degradation of Delta. Dev. Cell.

[78] Seto ES, Bellen HJ, Lloyd TE (2002). When cell biology meets development: endocytic regulation of signaling pathways. Genes Dev.

[79] Le Borgne R, Bardin A, Schweisguth F (2005). The roles of receptor and ligand endocytosis in regulating Notch signaling. Development.

[80] Parks AL, Klueg KM, Stout JR, Muskavitch MA (2000). Ligand endocytosis drives receptor dissociation and activation in the Notch pathway. Development.

[81] Frise E, Knoblich JA, Younger-Shepherd S, Jan LY, Jan YN (1996). The *Drosophila* Numb protein inhibits signaling of the Notch receptor during cell-cell interaction in sensory organ lineage. Proc. Natl. Acad. Sci. USA.

[82] Santolini E, Puri C, Salcini AE, Gagliani MC, Pelicci PG, Tacchetti C, Di Fiore PP (2000). Numb is an endocytic protein. J. Cell Biol.

[83] Berdnik D, Torok T, Gonzalez-Gaitan M, Knoblich JA (2002). The endocytic protein alpha-Adaptin is required for Numb-mediated asymmetric cell division in *Drosophila*. Dev. Cell.

[84] Jafar-Nejad H, Norga K, Bellen H (2002). Numb: "Adapting" notch for endocytosis. Dev. Cell.

[85] Lai EC, Posakony JW (1997). The Bearded box, a novel 3' UTR sequence motif, mediates negative post-transcriptional regulation of *Bearded* and *Enhancer of split* Complex gene expression. Development.

[86] Leviten MW, Lai EC, Posakony JW (1997). The *Drosophila* gene *Bearded* encodes a novel small protein and shares 3' UTR sequence motifs with multiple *Enhancer of split* complex genes. Development.

[87] Bardin AJ, Schweisguth F (2006). Bearded family members inhibit Neuralized-mediated endocytosis and signaling activity of Delta in *Drosophila*. Dev. Cell.

[88] Wurmbach E, Wech I, Preiss A (1999). *The Enhancer of split* complex of *Drosophila melanogaster* harbors three classes of Notch responsive genes. Mech. Dev.

[89] Lai EC, Bodner R, Kavaler J, Freschi G, Posakony JW (2000). Antagonism of Notch signaling activity by members of a novel protein family encoded by the *Bearded* and *Enhancer of split* gene complexes. Development.

[90] Petcherski AG, Kimble J (2000). Mastermind is a putative activator for Notch. Curr. Biol.

[91] Mumm JS, Kopan R (2000). Notch signaling: from the outside in. Dev. Biol.

[92] Castro B, Barolo S, Bailey AM, Posakony JW (2005). Lateral inhibition in proneural clusters: cis-regulatory logic and default repression by *Suppressor of Hairless*. Development.

[93] Maier D (2006). Hairless: the ignored antagonist of the Notch signalling pathway. Hereditas.

[94] Lyman DF, Yedvobnick B (1995). *Drosophila* Notch receptor activity suppresses Hairless function during adult external sensory organ development. Genetics.

[95] Schweisguth F (1995). *Suppressor of Hairless* is required for signal reception during lateral inhibition in the *Drosophila* pupal notum. Development.

[96] Bailey AM, Posakony JW (1995). Suppressor of Hairless directly activates transcription of *Enhancer of split complex* genes in response to Notch receptor activity. Genes Dev.

[97] Schweisguth F, Nero P, Posakony JW (1994). The sequence similarity of the *Drosophila* Suppressor of Hairless protein to the integrase domain has no functional significance *in vivo*. Dev. Biol.

[98] Morel V, Lecourtois M, Massiani O, Maier D, Preiss A, Schweisguth F (2001). Transcriptional repression by Suppressor of Hairless involves the binding of a Hairless-dCtBP complex in *Drosophila*. Curr. Biol.

[99] Barolo S, Walker RG, Polyanovsky AD, Freschi G, Keil T, Posakony JW (2000). A Notch-independent activity of *Suppressor of Hairless* is required for normal mechanoreceptor physiology. Cell.

[100] Barolo S, Stone T, Bang AG, Posakony JW (2002). Default repression and Notch signaling: Hairless acts as an adaptor to recruit the corepressors Groucho and dCtBP to Suppressor of Hairless. Genes Dev.

[101] Lai EC (2002). Keeping a good pathway down: transcriptional repression of Notch pathway target genes by CSL proteins. EMBO Rep.

[102] Hasson P, Egoz N, Winkler C, Volohonsky G, Jia S, Dinur T, Volk T, Courey AJ, Paroush Z (2005). EGFR signaling attenuates Groucho-dependent repression to antagonize Notch transcriptional output. Nat. Genet.

[103] Hasson P, Paroush Z (2006). Crosstalk between the EGFR and other signalling pathways at the level of the global transcriptional corepressor Groucho/TLE. Br. J. Cancer.

[104] Busseau I, Diederich RJ, Xu T, Artavanis-Tsakonas S (1994). A member of the Notch group of interacting loci, *deltex* encodes a cytoplasmic basic protein. Genetics.

[105] Diederich RJ, Matsuno K, Hing H, Artavanis-Tsakonas S (1994). Cytosolic interaction between deltex and Notch ankyrin repeats implicates deltex in the Notch signaling pathway. Development.

[106] Matsuno K, Diederich RJ, Go MJ, Blaumueller CM, Artavanis-Tsakonas S (1995). Deltex acts as a positive regulator of Notch signaling through interactions with the Notch ankyrin repeats. Development.

[107] Hori K, Fostier M, Ito M, Fuwa TJ, Go MJ, Okano H, Baron M, Matsuno K (2004). *Drosophila* Deltex mediates suppressor of Hairless-independent and late-endosomal activation of Notch signaling. Development.

[108] Ye Y, Lukinova N, Fortini ME (1999). Neurogenic phenotypes and altered Notch processing in *Drosophila presenilin* mutants. Nature.

[109] Portin P (2002). General outlines of the molecular genetics of the Notch signalling pathway in *Drosophila melanogaster*: a review. Hereditas.

[110] Pavlopoulos E, Pitsouli C, Klueg KM, Muskavitch MA, Moschonas NK, Delidakis C (2001). *neuralized* encodes a peripheral membrane protein involved in Delta signaling and endocytosis. Dev. Cell.

[111] Mlodzik M, Baker NE, Rubin GM (1990). Isolation and expression of *scabrous*, a gene regulating neurogenesis in *Drosophila*. Genes Dev.

[112] Renaud O, Simpson P (2001). *scabrous* modifies epithelial cell adhesion and extends the range of lateral signalling during development of the spaced bristle pattern in *Drosophila*. Dev. Biol.

[113] Hu X, Lee EC, Baker NE (1995). Molecular analysis of *scabrous* mutant alleles from *Drosophila melanogaster* indicates a secreted protein with two functional domains. Genetics.

[114] Knoblich JA, Jan LY, Jan YN (1995). Asymmetric segregation of Numb and Prospero during cell division. Nature.

[115] Knoblich JA, Jan LY, Jan YN (1997). The N terminus of the *Drosophila* Numb protein directs membrane association and actin-dependent asymmetric localization. Proc. Natl. Acad. Sci. USA.

[116] Okabe M, Imai T, Kurusu M, Hiromi Y, Okano H (2001). Translational repression determines a neuronal potential in *Drosophila* asymmetric cell division. Nature.

[117] Badenhorst P, Finch JT, Travers AA (2002). Tramtrack co-operates to prevent inappropriate neural development in *Drosophila*. Mech. Dev.

[118] Sakakibara S, Okano H (1997). Expression of neural RNA-binding proteins in the postnatal CNS: implications of their roles in neuronal and glial cell development. J. Neurosci.

[119] Okano H, Imai T, Okabe M (2002). Musashi: a translational regulator of cell fate. J. Cell Sci.

[120] Hirata J, Nakagoshi H, Nabeshima Y, Matsuzaki F (1995). Asymmetric segregation of the homeodomain protein Prospero during *Drosophila* development. Nature.

[121] Ryter JM, Doe CQ, Matthews BW (2002). Structure of the DNA binding region of prospero reveals a novel homeo-prospero domain. Structure.

[122] Yousef MS, Matthews BW (2005). Structural basis of Prospero-DNA interaction: implications for transcription regulation in developing cells. Structure.

[123] Bi X, Kajava AV, Jones T, Demidenko ZN, Mortin MA (2003). The carboxy terminus of Prospero regulates its subcellular localization. Mol. Cell Biol.

[124] Manning L, Doe CQ (1999). Prospero distinguishes sibling cell fate without asymmetric localization in the *Drosophila* adult external sense organ lineage. Development.

[125] Lai EC (2002). Micro RNAs are complementary to 3' UTR sequence motifs that mediate negative post-transcriptional regulation. Nat. Genet.

[126] Enright AJ, John B, Gaul U, Tuschl T, Sander C, Marks DS (2003). MicroRNA targets in Drosophila. Genome Biol.

[127] Stark A, Brennecke J, Russell RB, Cohen SM (2003). Identification of Drosophila MicroRNA targets. PLoS Biol.

[128] Li Y, Wang F, Lee JA, Gao FB (2006). MicroRNA-9a ensures the precise specification of sensory organ precursors in Drosophila. Genes Dev.

